# Coping styles, adversity quotient, and resilience among Chinese nursing students during clinical placement

**DOI:** 10.3389/fpsyg.2026.1775045

**Published:** 2026-03-25

**Authors:** Jiafang Xiao, Violeta Lopez, Taotao Zhang, Hongmei Wang

**Affiliations:** 1 School of Nursing, Hubei University of Medicine, Shiyan, China; 2 International Institute of Health Sciences, Colombo, Sri Lanka; 3 School of Health, University of New England, Armidale, NSW, Australia; 4 School of Nursing and Allied Medical Sciences, Holy Angel University, Angeles, Philippines

**Keywords:** adversity quotient, Chinese nursing students, clinical placement, coping style, resilience

## Abstract

**Background:**

This study examined the experiences of Chinese undergraduate nursing students and identified factors associated with coping style, adversity quotient (AQ), and resilience during clinical placement.

**Methods:**

A cross-sectional study was conducted across three hospitals in Wuhan, China. A total of 343 undergraduate nursing students completed the Medical Staff Resilience Scale (MSRS), the Simple Coping Style Questionnaire (SCSQ), and the Adversity Quotient Scale (AQS) during the final 2 weeks of a 40-week clinical practice placement.

**Results:**

Resilience was positively correlated with adversity quotient and coping (*r* = 0.796, *r* = 0.820, *p* < 0.01). Linear regression analysis showed that gender (*β* = 0.224), coping style (*β* = 0.398), and adversity quotient (*β* = 0.439) were significant influencing factors of nursing students’ resilience, with statistically significant differences (*p* < 0.05). Together, these variables accounted for 71% of the total variance in resilience.

**Conclusion:**

Nursing students with higher levels of coping and adversity quotient were more resilient in dealing with adversity during clinical placement. Female nursing students demonstrated higher levels of resilience than their male counterparts in the same clinical placement context. Therefore, this study supports the need to integrate resilience and coping strategies into nursing education, especially for male students.

## Introduction

Resilience is a widely used term that denotes an individual’s capacity to cope with and adapt effectively in the face of stress, trauma, or adversity. It reflects psychological flexibility, emotional regulation, and the ability to maintain functional wellbeing despite significant life challenges (Aburn et al., 2016; Padmanabhanunni et al., 2022). In recent years, resilience has garnered increasing attention in nursing literature, as it is recognized as a critical determinant of nurses’ capacity to navigate the intense pressures, ethical dilemmas, and emotional demands inherent in clinical practice (Dutra et al., 2018; Chipps et al., 2025). With the increasing complexity of healthcare environments and escalating workloads, the mental wellbeing of clinical nurses, and, by extension, that of nursing students undergoing clinical training, has become a pressing concern (Marshman et al., 2022).

While existing research consistently links higher resilience with better psychological outcomes and professional retention in nursing (Haeffel and Vargas, 2011; Osaimi et al., 2023), a significant gap remains in understanding how resilience develops, particularly among student nurses. Nursing students, as novices in high-stakes clinical settings, are especially vulnerable. They must simultaneously acquire technical competencies, develop professional identities, and manage interpersonal dynamics while also confronting emotionally taxing situations such as patient suffering, death, and performance evaluation (Lekan et al., 2018). Studies globally have found that the stress levels of nursing students are generally high, acting as a significant predictor of their wellbeing and self-preservation (Spurr et al., 2020; Tanji et al., 2020; Porcar et al., 2019). These stressors often trigger anxiety and frustration, and given that their coping repertoires are still emerging, they are at a heightened risk of burnout compared to experienced nurses (Hand et al., 2021). Therefore, a lack of sufficient psychological resources will not only compromise their ability to deliver safe, empathetic, and effective patient care but also further impair their mental health. This has led researchers to advocate for enhanced mental health support and educational interventions aimed specifically at this group (Grimes et al., 2020; Çalışkan et al., 2025).

Although the concept of resilience has received widespread attention and has been well studied globally, research on this topic in China has a relatively short history but has grown rapidly in popularity (Li, 2020). The Chinese government has demonstrated a strong commitment to student mental health through initiatives such as the National Standards for Undergraduate Nursing Education Quality (Ministry of Education of the People’s Republic of China, 2018) and the National Mental Health Screening and Promotion Program (National Health Commission of the People’s Republic of China, 2022). This has led to a proliferation of research on nursing students’ mental health in China. Early findings indicate that Chinese nursing students may face unique challenges. For instance, their resilience scores have been found to be significantly lower than domestic norms and those of their international peers—a finding potentially linked to factors such as family expectations influencing career choice rather than personal agency (Chen et al., 2020; Li, 2020). Furthermore, existing studies on resilience among Chinese nursing students have focused primarily on campus-based participants, creating a critical knowledge gap regarding those undergoing clinical placement. As a core 40-week internship component of the Chinese undergraduate nursing curriculum, clinical placements provide an essential setting for nursing students to translate theoretical knowledge into real-world practice (Guo et al., 2021).

To understand how nursing students navigate this pivotal period, it is essential to examine the key psychological constructs that underpin their ability to manage stress. Coping style refers to the cognitive and behavioral strategies individuals employ to manage stress and is primarily classified into two types: Positive coping and passive coping (Patterson and McCubbin, 1987). Positive coping strategies, such as problem-solving and seeking support, contribute to better emotional health (Huang et al., 2021), whereas negative strategies, including self-blame and avoidance, have harmful effects on emotional states (Keener et al., 2021). Meanwhile, adversity quotient (AQ) is defined as an individual’s perceived capacity to endure, navigate, and grow from challenging situations, thereby reflecting motivational persistence and perceived control in the face of adversity (Stoltz, 1997). Research indicates that nursing students’ levels of autonomy and engagement in healthy behaviors are positively associated with their AQ (Suksatan et al., 2021; Safi'i et al., 2021). Currently, there are studies on the relationship between coping style and resilience and the relationship between AQ and resilience (Xu and Yang, 2023; Saxena and Rathore, 2025); however, few have examined all three constructs together within the context of nursing clinical placement.

This omission is significant because resilience does not operate in isolation; instead, it likely arises from the dynamic interplay between how students perceive adversity (AQ), the strategies they employ to deal with it (coping style), and their subsequent ability to recover and flourish (resilience) (Patterson and McCubbin, 1987; Stoltz, 1997; Biggs et al., 2017). Without an integrated understanding, educational interventions may remain fragmented and less effective. Therefore, this study addresses the critical lack of integrated empirical evidence regarding how coping style and AQ jointly shape resilience among Chinese undergraduate nursing students during clinical placements. Clarifying these relationships is essential for developing targeted, evidence-based educational strategies to foster psychological resilience among nursing students during one of the most formative and stressful stages of their professional training (Çalışkan et al., 2025).

## Methods

### Design

A cross-sectional survey design was employed in this descriptive study to analyze population characteristics at a specific time point and examine relationships between variables (Wang and Cheng, 2020). However, generalizability may be limited due to potential sampling bias, as participants were not randomly selected from the broader population of nursing students undergoing clinical placement.

The sample size was calculated for multivariate analysis (MVA) using the assumption of 10 to 20 participants per independent variable. Our study considered a total of 14 factors, including four general demographic variables of nursing students and 10 dimensions from each scale, with an additional 10% added to account for potential loss to follow-up or invalid questionnaires (*n* = 14 × (10–20) × (1 + 10%) = 154–308). Therefore, a sample size of 343 was determined to be required to achieve statistical significance at a 95% confidence interval (Wang et al., 2022). The Strengthening the Reporting of Observational Studies in Epidemiology (STROBE) checklist was used to report the study data.

### Setting

In late March 2024, during the final 2 weeks of their internship, 343 undergraduate nursing students from three hospitals in Wuhan City, Hubei Province, China, were recruited as study participants. The clinical placement period for all participants extended from June 2023 to March 2024. The three hospitals included in this study are all university-affiliated hospitals and were selected due to their long-term, stable cooperative relationship with our nursing school and their representativeness of clinical teaching settings, ensuring the reliability of the study participants.

Our study’s inclusion criteria included all fourth-year undergraduate nursing students undertaking a 40-week internship in Grade A general city hospitals (>1,000 beds) in Wuhan City, China. The exclusion criteria were nursing students completing their clinical practice at smaller hospitals and those with preexisting mental or physical illnesses or disabilities, which were determined through a review of medical records provided by the university health center. This approach was chosen to ensure more accurate identification of preexisting conditions than relying on self-report. Data were collected using Questionnaire Star, an online questionnaire platform widely used in academic research. During data collection, the researchers’ e-mail addresses were provided so that participants could contact them with any questions. A pilot test was conducted with a small sample of 10 nursing students before formal data collection to ensure that the questionnaire was clear and understandable. This pilot sample was not included in the final study sample. No financial incentives or monetary rewards were provided for participation. Participation was entirely voluntary.

## Outcome measures

### Measures

#### Demographic characteristics

We obtained participants’ information on gender, family status, age, and clinical practice department.

#### Chinese medical staff resilience scale (C-MSRS)

The 18-item Chinese Medical Staff Resilience Scale (C-MSRS) (Zhu et al., 2016) consists of four subscales: Decision coping (6 items), interpersonal connection (4 items), rational thinking (4 items), and flexible adaptation (4 items). Responses to each item are rated on a 5-point Likert scale, ranging from 1 (low) to 5 (high) levels of resilience. Total scores on the C-MSRS range from 18 to 90, with higher scores indicating greater resilience. The internal consistency reliability of this C-MSRS was previously reported as Cronbach’s alpha = 0.90 (Zhu et al., 2016). In our study, the scale’s internal consistency reliability was Cronbach’s alpha = 0.92.

#### Chinese simple coping style questionnaire (C-SCSQ)

The 20-item Chinese Simple Coping Style Questionnaire (C-SCSQ) (Xie, 1998) comprises two subscales: positive coping style (12 items), with scores ranging from 0 to 3 for each item and a total score ranging from 0 to 36, and negative coping style (8 items), with scores ranging from 0 to 3 for each item and a total score ranging from 0 to 24. Higher total scores on either subscale indicate more frequent use of that coping style. When the scale was applied to nursing staff, the internal consistency of the C-SCSQ was acceptable, with Cronbach’s alpha of 0.84 for the positive coping style subscale and 0.82 for the negative coping style subscale. In our study, the C-SCSQ demonstrated excellent internal consistency, with Cronbach’s alpha values of 0.90 and 0.92 for the positive and negative coping style subscales, respectively.

#### Chinese adversity quotient scale (C-AQS)

The C-AQS (Li and Chen, 2008) consists of 30 scenarios, with two items per scenario, for a total of 60 items. Of these, 40 items represent adversity scenarios (scored items), while 20 items represent favorable scenarios (unscored and used solely for control purposes). The adversity scenarios are divided into four dimensions: Control, origin and ownership, reach, and endurance, with 10 items assigned to each dimension. A 5-point Likert scale is used for scoring, ranging from “1 = strongly disagree” to “5 = strongly agree.” The total score for the adversity portion ranges from 40 to 200, and each dimension score ranges from 10 to 50. Higher total scores indicate a greater adversity quotient and a stronger ability to cope with adversity. In Li and Chen’s study, the internal consistency reliability was reported as Cronbach’s alpha = 0.90 (Li and Chen, 2008). In our study, the internal consistency reliability of the C-AQS was also Cronbach’s alpha = 0.90.

### Data collection

This study was approved by the Science and Technology Ethics Committee of Hubei University of Medicine (Reference Number: 2024-RE-018). All potential participants were provided with written information about the study and were informed of their right to withdraw at any time. Consent was obtained, and electronic questionnaires were sent to participants’ mobile devices for data collection. Responses were anonymized and collated using unique identifiers. All questionnaires were checked for validity and completeness. The completed questionnaires were stored in a password-protected repository to ensure the confidentiality and anonymity of participants. Only the research team has access to these files.

### Statistical analysis

All data were analyzed using SPSS 26.0 to explore factors associated with resilience among undergraduate nursing students during clinical practice. Measurement data were analyzed using the *t*-test. Ordinal data were analyzed using one-way analysis of variance (ANOVA) and the chi-squared test. The significance level was set at *α* = 0.05, and a *p*-value of < 0.05 was considered statistically significant (Draxler, 2010).

## Results

### Demographic characteristics of nursing students

A total of 343 undergraduate nursing students fully completed all questionnaires. There were 106 (31%) male and 237 (69%) female nursing students, with ages ranging from 20 to 24 years. Most clinical placements were in medical wards (22.7%), surgical wards (19.5%), and gynecology and obstetrics wards (18.1). Family income was categorized into quartiles, ranging from 17 students (5%) in the low-income group [<35,000 RMB (US$ 4,970)] to 29 students (8.5%) in the very high-income group [≥150,000 RMB (US$ 21,303)].

### Nursing students’ stress resilience

The mean total score on the C-MSRS was 65.16 ± 14.37. Mean scores for the subscales were as follows: Decision coping, 21.52 ± 5.55; interpersonal connection, 75 ± 3.56; rational thinking, 14.32 ± 3.78; and flexible adaptation, 14.57 ± 3.53. Among the subscales, interpersonal connection had the highest mean score (Table 1).

**Table 1:** Nursing students’ stress resilience, simple coping style, and adversity quotient scores.

Variables	Number of items	Score range	Score (m ± sd)	Average score (m ± sd)
Stress resilience				
Decision coping	6	9—44	21.52 ± 5.55	3.58 ± 0.92
Interpersonal connection	4	4—20	14.75 ± 3.56	3.68 ± 0.59
Rational thinking	4	5—20	14.32 ± 3.78	3.58 ± 0.63
Flexible adaptation	4	6—20	14.57 ± 3.53	3.64 ± 0.88
C-MSRS total score	18	31—90	65.16 ± 14.37	3.62 ± 0.78
Simple coping style				
Positive coping	12	4–36	24.27 ± 8.22	2.02 ± 0.68
Negative coping	8	0–24	14.9 ± 6.21	1.86 ± 0.77
Adversity quotient				
Control	10	10–50	35.98 ± 8.83	3.59 ± 0.88
Attribution	10	13–50	35.6 ± 8.91	3.59 ± 0.88
Extension	10	12–50	35.71 ± 8.86	3.57 ± 0.88
Endurance	10	16–50	35.80 ± 8.46	3.68 ± 0.84
C-AQS total score	40	72–200	143.09 ± 33.78	3.57 ± 0.84

### Nursing students’ simple coping style

The mean total score on the C-SCQ was 39.16 ± 13.6, with an average item score of 0.11 ± 0.03. Specifically, the mean positive coping score was 24.27 ± 8.22, and the mean negative coping score was 14.9 ± 6.21 (Table 1).

### Nursing students’ adversity quotient

The total mean score on the C-SQS was 143.09 ± 33.78, with an average item score of 3.57 ± 0.84. The mean scores for the C-SQS subscales were 35.98 ± 8.83 for control, 35.6 ± 8.91 for attribution, 35.71 ± 8.86 for extension, and 35.80 ± 8.46 for endurance (Table 1).

### Comparison of resilience scores and differences in general demographic characteristics among nursing students

One-way analysis of variance (ANOVA) was used to compare differences in stress resistance among nursing students across different demographic characteristics. The results indicated that stress resilience scores were not statistically significantly associated with demographic characteristics, including placement department, age, and family economic status (*p* > 0.05). However, stress resilience scores differed significantly between male and female nursing students (*p* < 0.05) (Table 2).

**Table 2:** One-way analysis of variance of stress resilience scores in nursing students by demographic characteristics (*n* = 343).

Variables	*n*	%	Stress resilience score (m ± sd)	*t*/*F*	*p*
Sex				13.208	0.00
Male	106	30.9	61.02 ± 15.42		
Female	237	69.1	67.02 ± 13.51		
Family income				0.451	0.71
<35,000 RMB	17	5	65.00 ± 14.16		
35,000–90,000 RMB	211	61.50	65.44 ± 13.96		
90,000–150,000 RMB	86	25.10	65.52 ± 14.66		
≥150,000 RMB	29	8.50	62.21 ± 16.87		
Age:				0.727	0.57
20	9	2.60	65.11 ± 8.87		
21	44	12.80	68.21 ± 7.62		
22	122	35.60	64.66 ± 13.97		
23	84	24.50	65.63 ± 15.88		
24	84	24.50	63.85 ± 16.43		
Placement department				0.396	0.94
Surgical ward	67.00	19.50	63.78 ± 14.70		
Medical ward	78.00	22.70	64.35 ± 13.23		
Pediatric ward	55.00	16.00	66.80 ± 15.26		
Gynecology and obstetrics ward	62.00	18.10	65.68 ± 15.10		
Emergency ward	56.00	16.30	66.54 ± 15.47		
Geriatrics ward	3.00	0.90	57.00 ± 13.23		
Operating room	8.00	2.30	68.00 ± 8.29		
Infectious disease ward	5.00	1.50	62.60 ± 13.69		
Oncology ward	5.00	1.50	66.80 ± 15.77		
Other departments	4.00	1.20	63.50 ± 9.17		

RMB = Chinese Yuan Renminbi 1,000 = US$ 138.

### Correlations between stress resilience and coping styles among nursing students

The total score and all subscales of stress resilience were significantly positively correlated with all subscales of the C-SCSQ (*p* < 0.01) (Table 3).

**Table 3:** Correlation analysis between stress resilience and simple coping style (*n* = 343).

Aspects	Stress resilience	Decision coping	Interpersonal connection	Rational thinking	Flexible adaptation
Positive coping	0.820**	0.677**	0.694**	0.756**	0.763**
Negative coping	0.678**	0.567**	0.511**	0.613**	0.698**

**At the 0.01 level (two-tailed), the correlation was significant.

### Correlations between stress resilience and adversity quotient among nursing students

The total score and all subscales of stress resilience were significantly positively correlated with all adversity quotient scores (*p* < 0.01) (Table 4).

**Table 4:** Correlation analysis between stress resilience and adversity quotient in nursing students.

Aspects	Adversity quotient	Control	Attribution	Extension	Endurance
Stress resistance	0.796**	0.763**	0.780**	0.762**	0.763**
Decision coping	0.664**	0.643**	0.656**	0.621**	0.641**
Interpersonal connection	0.641**	0.631**	0.622**	0.618**	0.600**
Rational thinking	0.734**	0.688**	0.725**	0.715**	0.701**
Flexible adaptation	0.765**	0.723**	0.743**	0.740**	0.741**

**At the 0.01 level (two-tailed), the correlation was significant.

### Factors affecting stress resilience among nursing students

In the multivariate regression analysis, gender, adversity quotient, and simple coping style were included as independent variables to identify factors associated with stress resilience as dependent variables. Then, we obtained the results of the linear regression analysis of stress resistance, as shown in Table 5.

**Table 5:** Linear regression analysis of nursing students’ stress resilience (*n* = 343).

Independent variable	*B*	SE	Beta	*t*	*p*
Constant	10.213	2.812		3.631	0.000
Gender	6.945	0.911	0.224	7.623	0.000
Simple coping style	0.421	0.083	0.398	5.040	0.000
Adversity quotient	0.187	0.034	0.439	5.545	0.000

*R*^2^ = 0.71.

## Discussion

### Gender differences in stress resilience among nursing students

Among the 343 nursing students in this study, gender was identified as a significant factor associated with stress resilience. Female students constituted the majority of participants and were found to have a higher level of stress resilience. This association may be linked to evidence indicating that Chinese male nursing students have a lower sense of professional identity in nursing compared to female nursing students (Zeabadi et al., 2021). Hardan et al. (2025) noted that male individuals perceived hospital work as “trivial” and “difficult” for meeting their personal career development needs. In addition, most male nursing students are vulnerable to the effects of traditional social and occupational prejudices. A key cultural factor contributing to this association is the long-standing perception in Chinese culture that nursing is a female-dominated profession, which can result in male nurses experiencing rejection from patients during nursing procedures (Mao et al., 2020). Consequently, male nursing students are more likely to experience emotional fluctuations and greater psychological pressure. This finding that female nursing students show higher resilience than their male counterparts contrasts with Western literature, which generally reports no significant gender differences or greater resilience among male nursing professionals (Labrague, 2024).

This discrepancy can be attributed to cultural variations in gender roles and occupational perceptions: In Western contexts, nursing has become increasingly gender-neutral, with male nurses gaining broader social acceptance and professional recognition, whereas traditional gender norms in China still cast nursing as a “feminine” occupation, placing additional psychological burdens on male practitioners. This cultural contrast highlights the importance of considering sociocultural contexts when interpreting gender-related differences in resilience (Gao et al., 2019).

### Strong correlations among resilience, coping styles, and adversity quotient

It is worth noting that the present study observed very strong correlations (*r* > 0.79) among resilience, coping styles, and adversity quotient. These strong correlations suggest potential construct overlap or multicollinearity, which warrants careful consideration when interpreting the results (Hair et al., 2019). The substantial correlations among these three constructs may indicate that they share common underlying psychological dimensions. Resilience is conceptualized as the interactive influence of psychological characteristics within the context of the stress process, encompassing an individual’s capacity to adapt positively in the face of adversity (Bergeman and Nelson, 2024). Similarly, the adversity quotient (AQ), originally proposed by Stoltz (1997), assesses an individual’s response pattern when facing setbacks and difficulties, comprising four core dimensions (Control, Origin, Reach, and Endurance). Coping styles, particularly positive coping strategies, represent the cognitive and behavioral efforts individuals make to manage stressful situations (Alonso-Tapia et al., 2019). The conceptual overlap among these constructs is theoretically plausible. Positive coping may function as an inherent component of both resilience and adversity quotient, as all three constructs fundamentally reflect aspects of psychological adaptability and stress management capacity (Nimo et al., 2025). For instance, individuals with higher resilience tend to use more problem-focused coping strategies (Rodríguez-Planas and Secor, 2026), while those with higher AQ demonstrate greater perceived control over adverse events and are more likely to engage in active coping behaviors (Lee et al., 2025). This shared variance may explain the strong correlations observed in our sample.

From a methodological perspective, multicollinearity among predictor variables can lead to inflated or unstable estimates of the relationships between individual constructs and outcome variables (Hair et al., 2017). When constructs are highly correlated, it becomes challenging to disentangle their unique contributions to predicted outcomes, potentially affecting the interpretability of regression coefficients and path estimates. In the present study, this issue may have influenced the estimated effects of these psychological constructs on variables such as gender differences or clinical performance outcomes.

Future research could address this issue through several approaches. First, confirmatory factor analysis could be employed to examine the discriminant validity of these three constructs, clarifying whether they measure distinct theoretical concepts. Second, qualitative research methods could be utilized to explore the unique manifestations and interactions of resilience, coping styles, and adversity quotient among nursing students during clinical practice. For instance, Aryuwat et al. (2024), in a qualitative study of Thai nursing students, identified two core themes in the formation of resilience: “experiences of vulnerability” and “experiences of meaningfulness,” with social support and positive transformation playing crucial roles. Similar methodological approaches may help elucidate the unique and overlapping dimensions of these three constructs within the context of nursing education.

### Implications for nursing education

Gender differences in stress resilience and the strong correlations among resilience, coping styles, and adversity quotient (AQ) in our sample highlight the need for targeted strategies in nursing education to enhance stress resilience in nursing students, especially among male nursing students, and to promote their overall psychological adaptability.

Primarily, nursing education institutions should establish a gender-sensitive resilience education system that fully considers the differences in stress resilience and its influencing factors between male and female nursing students (Ye et al., 2025; Jarrad et al., 2025). Given that male nursing students in China experience lower professional identity, higher psychological pressure, and greater susceptibility to traditional occupational prejudices (Wu et al., 2023), nursing education should focus on addressing occupational stereotypes and enhancing their professional recognition (Al-Hammouri et al., 2024). This can be achieved by integrating content related to the value of male nurses in clinical practice into nursing courses, inviting exemplary male clinical nurses to share their career experiences as role models (Azadian et al., 2024), and organizing thematic discussions to address social prejudices against male nurses. For female nursing students, although they exhibit higher stress resilience (Jarrad et al., 2025), nursing education should still emphasize the cultivation of their psychological defense mechanisms to help prevent excessive internal depletion in the face of clinical stress.

Furthermore, given the strong correlations among resilience, coping styles, and AQ, nursing education should implement an integrated training model to simultaneously improve these three interrelated psychological constructs. Since positive coping styles are an inherent component of both resilience and AQ (Fletcher and Sarkar, 2013; Stoltz, 1997), nursing educators can systematically incorporate stress management and coping skills training into the curriculum. Specific measures include designing scenario-based simulation exercises that simulate clinical setbacks and pressure situations, guiding students to practice problem-focused coping strategies (Issac et al., 2026), and helping them develop a positive response pattern when facing difficulties—consistent with the core dimensions of AQ (Control, Origin, Reach, and Endurance) (Stoltz, 1997). Furthermore, special courses or workshops on AQ enhancement can be set up to help students cultivate rational cognitive attitudes toward adversity, thereby improving their overall psychological adaptability (Drury Young et al., 2024).

In addition to the training model mentioned above, nursing education should strengthen the integration of theoretical teaching and clinical practice (Bester et al., 2024) and optimize the support system for nursing students during their clinical transition period (Cordon et al., 2025). The long-standing perception of nursing as a female-dominated profession often results in male nursing students experiencing patient rejection during clinical procedures (Hardan et al., 2025), which increases their psychological stress. Therefore, clinical mentors should be trained to provide gender-sensitive guidance: For male nursing students, mentors can offer targeted psychological support and practical guidance to help them handle interpersonal conflicts and patient rejection; for all students, mentors should timely identify their emotional fluctuations and guide them in applying the coping strategies and resilience skills acquired in theoretical courses to clinical practice.

Moreover, nursing education institutions should improve the professional quality of the teaching team and build a supportive educational environment to complement the aforementioned measures. Targeted training that addresses gender differences in psychological resilience and integrates approaches to developing resilience, coping styles, and AQ can better equip them to meet the diverse needs of students (Mayor-Silva et al., 2024). Meanwhile, a sound psychological counseling system should be established to provide timely psychological intervention for nursing students with emotional distress, especially male students who are more prone to psychological pressure (Campbell and Mazurek Melnyk, 2025). In addition, extracurricular activities, such as team building and psychological quality development, can be organized to enhance students’ social support and self-confidence, which are crucial for improving stress resilience (University of Hawaii at Manoa, 2025).

Finally, nursing education should pay attention to the cultural context of nursing practice in China. Unlike Western countries where nursing has become increasingly gender-neutral, traditional gender norms in China still impose additional psychological burdens on male nursing students (Xu et al., 2024). Therefore, nursing education should not only learn from advanced international experiences but also integrate the specific cultural and social context of China to formulate more targeted and feasible educational strategies (Li et al., 2025). This includes enhancing public awareness and education about the professional value of nursing to reduce social prejudices against male nurses, thereby creating a more inclusive professional environment for nursing students and laying a solid foundation for improving their stress resilience.

## Limitations

This study has several limitations. First, the participants were exclusively Chinese undergraduate nursing students aged 20–24 years, resulting in a relatively narrow sample, which limits the generalizability of the findings to other nursing populations or cultural groups. Second, convenience sampling, while cost-effective and appropriate for exploratory research, may introduce selection bias, such that the findings are not fully representative of the target population, thereby limiting generalizability. Although we attempted to reduce bias through online surveys, this recruitment strategy may have led to the overrepresentation of more technologically adept individuals, further limiting sample representativeness and the generalizability of the results. In addition, other unmeasured factors may influence stress resilience and coping styles when students face adversity during clinical placements. Future studies should expand the research scope and examine a wider range of potential influencing factors.

## Conclusion

Gender, coping styles, and adversity quotient were identified as significant factors influencing stress resilience among nursing students. Students with higher levels of simple coping and adversity quotient demonstrated greater stress resilience. Furthermore, female nursing students exhibited higher resilience than their male counterparts, suggesting the need for gender-specific interventions. Therefore, to support the sustainable and healthy career development of nursing students, nursing educators, academic institutions, and clinical hospitals should pay closer attention to students’ psychological wellbeing and provide necessary humanistic care.

## Data Availability

The datasets presented in this study can be found in online repositories. The names of the repository/repositories and accession number(s) can be found at https://pan.baidu.com/s/1PsDAXxjrcAnCTgMIRYiXAA?pwd=7csk. Extraction code:7cs.
